# Psychometric properties of the patient-reported outcomes measurement information system scale v1.2: global health (PROMIS-GH) in a Dutch general population

**DOI:** 10.1186/s12955-021-01855-0

**Published:** 2021-09-27

**Authors:** Leonardo Pellicciari, Alessandro Chiarotto, Emanuele Giusti, Martine H. P. Crins, Leo D. Roorda, Caroline B. Terwee

**Affiliations:** 1 Neurorehabilitation Research Laboratory, IRCCS San Raffaele Roma, Rome, Italy; 2 Department of Health Sciences, Amsterdam Movement Sciences Research Institute, VU University, Amsterdam, The Netherlands; 3 Department of General Practice, Erasmus MC, , University Medical Center, Rotterdam, The Netherlands; 4 Psychology Research Laboratory, IRCCS Istituto Auxologico Italiano, Milan, Italy; 5 Department of Psychology, Catholic University of the Sacred Heart, Milan, Italy; 6 Amsterdam Rehabilitation Research Center | Reade, Amsterdam, The Netherlands; 7 Zuyderland MC Department of Quality and Safety, Amsterdam, The Netherlands; 8 Department of Epidemiology and Data Science, Amsterdam UMC, Vrije Universiteit Amsterdam, Amsterdam Public Health Research Institute, de Boelelaan 1089a, 1081 HV Amsterdam, The Netherlands

## Abstract

**Purpose:**

To assess the psychometric properties of the Dutch-Flemish Patient-Reported Outcome Measurement Information System Scale v1.2 – Global Health (PROMIS-GH).

**Methods:**

The PROMIS-GH (also referred to as PROMIS-10) was administered to 4370 persons from the Dutch general population. Unidimensionality (CFI ≥ 0.95; TLI ≥ 0.95; RMSEA ≤ 0.06; SRMR ≤ 0.08), local independence (residual correlations < 0.20), monotonicity (H > 0.30), model fit with the Graded Response Model (GRM, *p* < 0.001), internal consistency (alpha > 0.75), precision (total score information across the latent trait), measurement invariance (no Differential Item Functioning [DIF]), and cross-cultural validity (no DIF for language, Dutch vs. United States English) of its subscales, composed of four items each, Global Mental Health (GMH) and Global Physical Health (GPH), were assessed.

**Results:**

Confirmatory factor analyses, on both subscales, revealed slight departures from unidimensionality for GMH (CFI = 0.98; TLI = 0.95, RMSEA = 0.22; SRMR = 0.04) and GPH (CFI = 0.99; TLI = 0.97; RMSEA = 0.12; SRMR = 0.03). Local independence, monotonicity, GRM model fit, internal consistency, precision and cross-cultural validity were supported. However, Global10 (emotional problems) showed misfit on the GMH subscale, while Global08 (fatigue) presented DIF for age.

**Conclusion:**

The psychometric properties of the PROMIS-GH in the Dutch population were considered acceptable. Sufficient local independence, monotonicity, GRM fit, internal consistency, measurement invariance and cross-cultural validity were found. If future studies find similar results, structural validity of the GMH could be enhanced by improving or replacing Global10 (emotional problems).

**Supplementary Information:**

The online version contains supplementary material available at 10.1186/s12955-021-01855-0.

## Introduction

Health-related quality of life (HRQoL) refers to the ‘‘physical, psychological, and social domains of health, seen as distinct areas that are influenced by a person’s experiences, beliefs, expectations, and perceptions’’ [1]. HRQoL measures are increasingly used as outcome indicators to evaluate outcomes of health care and to assess the effectiveness of intervention programs in the general population and in patients with specific diseases. HRQoL is included as a core outcome (construct) in many core outcome sets, such as those for patients with back pain [2], aphasia [3], cardiac arrest [4], psoriatic arthritis [5], prostate cancer [6], hip and knee osteoarthritis [7], whiplash associated disorders [8], and in many Standard Sets of the International Consortium of Health Outcomes Measurement (ICHOM) [9]. Sound HRQoL measurement is crucial to ensure that clinicians and researchers evaluate HRQoL in an optimal way, which is achieved when reliable and valid measurement instruments are being used [10].

The Patient-Reported Outcomes Measurement Information System (PROMIS®) initiative [11] was established to measure HRQoL in the general population and in patients with any kind of disease. Item banks were developed using Item Response Theory (IRT) methods, which can be administered as short forms or computerized adaptive tests. The item banks measure a wide range of physical, mental and social health domains [12]. The PROMIS initiative developed, amongst others, the PROMIS Scale Global Health (PROMIS-GH), representing five core health domains (physical health, pain, fatigue, mental health, social health, and overall health) [13]. The PROMIS-GH consists of ten items and is also referred to as PROMIS-10. The psychometric properties of the PROMIS-GH have been assessed through factor analyses in United States (US) general population. Results indicated a 2-factor structure which led to the development of two subscales: Global Mental Health (GMH) and Global Physical Health (GPH). Both subscales demonstrated good internal consistency (α = 0.81 and 0.86 for GPH and GMH, respectively). Moreover, both subscales fitted an IRT-model, enabling calculation of IRT-based scores [13]. Katzan and Lapin [14] confirmed, in stroke patients, the 2-factor structure and the good internal consistency (α = 0.82 and 0.88 for GPH and GMH, respectively). The PROMIS-GH was recommended by panels of international experts as a brief measure of HRQoL, e.g., for patients with low back pain and stroke [15, 16], and was recently included in the ICHOM overall adult health Standard Set to be measured in all patients with or without any disease [9].

To our knowledge, no studies assessed the psychometric properties of the PROMIS-GH in a general population sample outside the US [17]. Also, no studies so far evaluated measurement invariance for language (or cross-cultural validity) which is a key property for international comparisons. Therefore, the aims of this study were to assess the psychometric properties of the PROMIS-GH in a Dutch general population sample, including an assessment of measurement invariance for language, and to provide recommendations for its use by clinicians and researchers.

## Methods

### Participants

Participants were recruited from an existing internet panel of the Dutch general population by a data collection company (Desan Research Solutions; certified for ISO-20252—market research and opinion research and ISO-27001—data security). The panel was provided by Global Market Insite (GMI). Panellists were recruited mainly through telephone and ads and banners on websites. Informed consent to become a panellist is ensured by GMI. For this particular study, panellists were recruited in 4 waves by an invitation from the panel host. Panelists receive “panel points” by participating in studies, which they can collect at regular intervals to receive a small amount of money, or—more often—a web voucher. For our study, panelists were recruited by an invitation from the panel host. The invitation mentioned the topic and length of the survey. By voluntarily responding to the invitation for this survey, panelists provided informed consent to participate in the study. All data collected were strictly anonymous, as the data collection company did not know the identity of the respondents, and the panel provider did not know what panelists responded to the survey.

The sample needed to be representative of the Dutch general population, according to data from Statistics Netherlands in 2016 (www.cbs.nl) (maximum of 2.5% deviation) with respect to distribution of age (18–40; 40–65; > 65), gender, education (low, middle, high), region (north, east, south, west), and ethnicity (native, first and second generation western immigrant, first and second generation non-western immigrant).No information was collected about the response rate. The Medical Ethics Review Committee of VU University Medical Center confirmed that the Medical Research Involving Human Subjects Act (WMO) does not apply to this study and that an official approval of this study by the committee was not required; the reason for this is that the test subjects are not subjected to any action and they are not imposed a mode of conduct, as laid down in the WMO.

In addition, we used data from the US PROMIS Wave 1 sample, obtained from the Health Measures Dataverse [12, 18], to study cross-cultural validity of the PROMIS-GH. The US data was also collected via a web-based survey to a national internet panel maintained by Polimetrix (now YouGovPolimetrix; see www.polimetrix.com).

### Procedures

This study was part of a larger initiative to assess the psychometric properties of eight full Dutch-Flemish PROMIS item banks and the PROMIS-GH in the Dutch general population [19, 20]. Four groups (three ≥ 1000 people and one ≥ 1300 people), were deemed necessary for item parameter estimation of these eight full item banks. The Dutch-Flemish v1.2 PROMIS-GH was administered to all four groups, in addition to one or more PROMIS banks. Participants were invited to complete all 10 items of the Dutch-Flemish PROMIS-GH through an online survey. Furthermore, subjects responded to general questions regarding their age, gender, educational level, region, and ethnicity.

### v1.2 PROMIS global health

The v1.2 PROMIS-GH consists of ten items [13]. Each item is scored on a 5-points scale, except Global07 which is scored on a 11-points numerical scale and recoded to a 5-points scale (as suggested by the PROMIS-GH Scoring Manual). Two items (Global08 and Global10) have reversed scoring and need to be recoded when calculating scores. Two total scores are calculated. The GMH score, addressing mental health, is calculated from four items: Global02 (overall quality of life), Global04 (mental health), Global05 (satisfaction with social activities) and Global10 (emotional problems). The GPH score, addressing physical health, is also calculated from four items: Global03 (physical health), Global06 (physical function), Global07 (pain intensity) and Global08 (fatigue).The remaining two items, Global01 (general health) and Global09 (ability to carry out social activities), do not contribute to the calculation of the total scores but can be used as single items. The total scores are calculated based on the original US IRT-model and expressed as T-scores with a mean ± standard deviation of 50 ± 10 in the US general population. Scores can be calculated using an online scoring service provided by the US Assessment Center [21] or by calculating raw summed scores and converting them to a T-score, using a conversion Table presented in the PROMIS-GH Scoring Manual [22]. Higher scores indicate better global mental/physical health. The v1.2 PROMIS-GH was translated into Dutch-Flemish using the FACIT translation methodology adopted by PROMIS and approved by the PROMIS language coordinator [23]. The English v1.2 PROMIS-GH can be downloaded from www.healthmeasures.net [24], after accepting the terms of agreement. Other language versions can be obtained from the Health Measures group or from country-specific PROMIS National Centers.

### Statistical analysis

Descriptive statistics were used to describe the socio-demographic characteristics of the sample and the distributions of the items. Table 1 provides an overview of the research questions both from a user perspective (clinicians or researchers who intend to apply the measure) and a psychometric perspective (researchers that investigate the psychometric properties of the measure), and include the specific psychometric properties studied, the statistical indexes calculated, the criteria for their interpretation, and the software packages used. The analysed psychometric properties of the PROMIS-GH encompass the PROMIS analyses plan [25].

**Table 1:** Summary of the research questions, the psychometric properties studied, the statistical analyses applied, and the results for the PROMIS Global Mental Health subscale (4 items) and the PROMIS Global Physical Health subscale (4 items) in the total Dutch general population sample (N = 4370)

Research questions from a users’ perspective	Research questions from a psychometric perspective	Psychometric property	Analyses per subscale	Statistic	Criteria	Reference	Software package with reference	Results	
								*GMH*	*GPH*
1. Is it legitimate to calculate IRT-based score for this measure?	Do the items assess only one construct?	Unidimensionality^a^	CFA	CFI	≥ 0.95	[44]	Mplus software (version 6.0) [45]	0.98	0.99
				TLI	≥ 0.95	[44]		0.95	0.97
				RMSEA	≤ 0.06	[44]		**0.22**	**0.12**
				SRMR	≤ 0.08	[44]		0.04	0.03
			Exploratory Bifactor Analysis	ECV	> 0.70	[46]	R package psych (version 1.7.8) [47]	0.80	0.71
				ωH	> 0.80	[48]		**0.75**	**0.65**
	Do the items relate to the construct being measured only?	Local independence	Residual correlation matrix^b^	r	≤ 0.20	[25]	Mplus software (version 6.0) [49]	all r < 0.20	all r < 0.20
	Do the probabilities of higher responses to the items increase with increasing levels of the construct?	Monotonicity	Mokken scale analysis	H_i_	≥ 0.30	[50]	R-package Mokken (version 2.8.4) [51]	See Table 3	See Table 3
				H	> 0.50	[50]		0.60	0.54
				ICCs^c^	Graphic display	[52, 53]		See Fig. 1a	See Fig. 1b
	Can the relationship between the items and the construct be described using an IRT-model?	IRT-model fit	Logistic GRM model fit	S-X^2^ and *p* of the items	*p* ≥ 0.001*	[44, 45]	R-package mirt (version 3.3.2) [54]	See Table 3	See Table 3
2. Is this measure able to discriminate between different levels of the construct/trait?	Do the items have the ability to discriminate between different levels of the construct/trait?	Range of item discrimination	IRT-modelling	α^d^	> 1.0	[52]	R-package mirt (version 3.3.2) [54]	See Table 3	See Table 3
3. Does this measure cover the relevant range of the construct/trait?	Do the items cover the relevant range of the construct/trait?	Range of item difficulties	IRT-modelling	β^e^	N/A	[52]	R-package mirt (version 3.3.2) [54]	See Table 3	See Table 3
4. Is this measure reliable?	What is the overall precision of this measure in this sample?	Internal consistency	Internal consistency	Cronbach’s alpha	> 0.70	[55]	SPSS software. Version 21 for Windows	0.83	0.78
	What is the contribution of the individual items to this overall precision?	Internal consistency	Internal consistency	Cronbach’s alpha if item deleted	Reduction of totalalpha	[56]		See Table 3	See Table3
			Corrected item-to-total correlation	r_s_	≥ 0.40	[57]		See Table 3	See Table 3
	What is the precision of this measure at different levels of the construct/trait?	Precision	TIC and IIC	Graphic display		[52, 53]		See Fig. 2a	See Fig. 2b
5/6. Does this measure function in the same way in different (sub)groups?	Can this measure be used to compare (sub)groups in terms of demographic variables?	Measurement invariance	DIF^g^	Change in Mcfadden R2	> 0.02	[44, 58, 59]	R-package lordif (version 0.3–3) [58]	See Table 3	See Table 3
	Can this measure be used to compare the scores of English-speaking persons (who responded to its English original version) and Dutch-speaking persons (who responded to its Dutch-Flemish translation)?	Cross-cultural validity	DIF^g^	Change in Mcfadden R2	> 0.02	[44, 58, 59]	R-package lordif (version 0.3–3) [58]	See Table 3	See Table 3

α, Item Discrimination Parameters estimated under the Graded Response Model; β, Item Difficulty Parameters estimated under the Graded Response Model; CFA, Confirmatory Factor Analysis; CFI, Comparative Fit Index; DIF, Differential Item Functioning; ECV, Explained Common Variance; GMH, General Mental Health; GPH, General Physical Health; GRM, Graded Response Model; H, scalability coefficient for the scale; H_i_, scalability coefficient for the item; ICC, Item Characteristics Curve; IIC, Item Information Curve; IRT, Item Response Theory; N/A, not appropriate;p, p-value; r, residual correlation; r_s_, Spearman correlation coefficient; RMSEA, Root Means Square Error of Approximation; S-X^2^, item fit statistics under the Graded Response Model; SRMR, Standardized Root Mean Square Residual; TIC, Test Information Curve; TLI, Tucker Lewis Index; ωH, Omega-Hierarchical

Statistics values beyond the recommended cut-off presented in bold

The research questions have been formulated from an user perspective (the clinicians or researchers who intend to apply the measure) and from a psychometric perspective (the researchers that investigate the psychometric properties of a measure)

The numbers next to the questions refer to the numbers of the measurement property reported in the methods

^a^A confirmatory two-factor analysis on the entire Global Health measure was initially run in order to confirm the two-factor structure. Once the two-factor structure was confirmed, analyses were performed on each subscale separately to confirm their unidimensionality, i.e., a unidimensional CFA [60] (fitted using a mean- and variance-adjusted Weighted Least Squares estimator) and an Exploratory Bifactor Analysis [48] (performed using a Schmid-Leiman procedure [61])

^b^Resulting from the single factor CFA

**Fig. 1:**
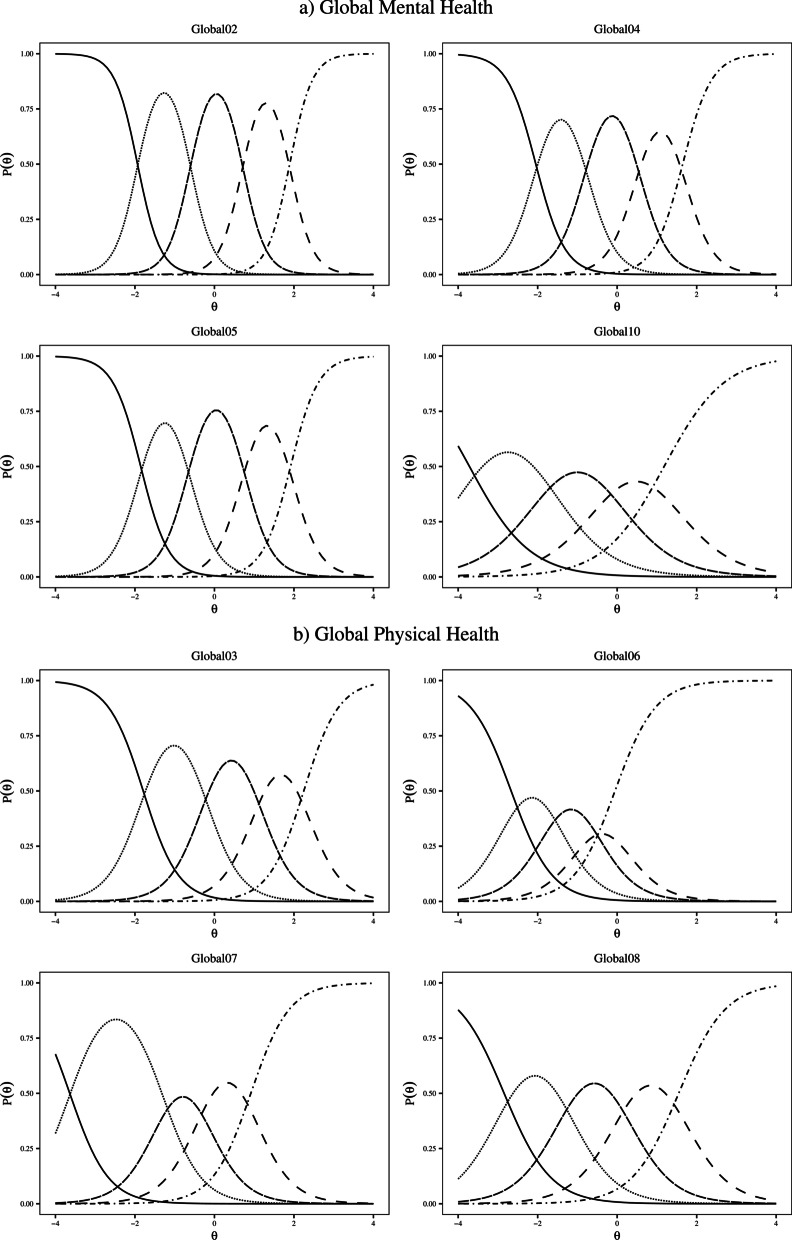
Item characteristic curves of the PROMIS Global Mental Health and Global Physical Health items (N = 4370)

^c^ICC graphs in Fig. 1, plotted for each item, visually illustrate the probability to select an item response across the level of ability

^d^ Item slopes indicate the ability of an item to discriminate between people with adjoining values on the latent trait

^e^Item thresholds refer to item difficulty, and locate the items along the latent trait

^f^ TICs and IICs plot the information across the latent trait at the total score-level or at item-level, respectively [52, 53]. In a unidimensional scale, the standard error (SE) is the reciprocal of the information (1/information) [62]; for each level of the latent trait and for each item, item information can be converted to a measure of reliability which can be interpreted as a Cronbach’s alpha using the following formula: 1-(SE) [52]; Information values of 10, 5 and 3.45 are therefore equal to internal reliability values of 0.90, 0.80, and 0.70 respectively [62]

^g^ A DIF [53]analysis was performed using a ordinal logistic regression framework. In the ordinal logistic regression framework, three regression models are compared to detect DIF, namely model 1 (item responses are predicted by the latent trait only), model 2 (item responses are predicted by the latent trait and group membership) and model 3 (item responses are predicted by the latent trait, group membership and the interaction between these two terms). Uniform and non-uniform DIF are present if model 2 has better fit than model 1 and if model 3 has better fit than model 2, respectively. The impact of DIF on item score and the total score was assessed by the visual display of ICCs per group and test characteristic curves per group, respectively

^*^Given the large sample size (N = 4370), we drew 10 mutually exclusive random sample of 473 subject each in order to minimize the chance to yield statistically significant results also for small fit differences

From a user perspective, for an IRT-derived measure, it is crucial to know whether:

1. It is legitimate to calculate IRT-based scores. This requires, from a psychometric perspective, that items meet the assumptions of an IRT-model (i.e., unidimensionality, local independence and monotonicity), and fit the underlying IRT-model (evidence for structural validity [26]). To study unidimensionality, both an exploratory and a confirmatory approach were used. First, a two-factor categorical Confirmatory Factor Analysis (CFA) on all items was performed, specifying two latent factors, namely mental health and physical health, allowing these factors to be correlated. Then, we checked if the two subscales could be considered as unidimensional scales and assessed potential modelling problems by performing two separate Exploratory Bifactor Analyses on each of the subscales. Finally, a unidimensional categorical CFA was performed on each subscale to evaluate if the data fit a unidimensional measurement model. Local dependence was investigated by examining the residual correlation matrix (≥ 0.20). Monotonicity was studied through Mokken scale analysis. Finally, the fit of the underlying IRT-model which results from the comparison between the expected item response functions under the Graded Response Model (GRM) and the observed item responses, was assessed using both fit indices and visual inspection of empirical plots.
  From a user perspective, it is also important that the measure:
2. Is able to discriminate between different levels of the construct (or latent variable or trait) and, as a consequence, is able to measure differences between persons or change within persons over time. This requires, from a psychometric perspective, that all item discrimination indexes, assessed using IRT modeling, are satisfactory.
3. Covers the relevant range of the construct, that is the range where future respondents ([healthy] persons or patients) are supposed to be located with respect to their health status. This requires, from a psychometric perspective, that the range of the item difficulties is acceptable. The range of item difficulties was assessed using IRT-modeling.
4. Is able to measure the total sample of respondents and respondents with different health states (standard error along the trait) reliably (or precisely). This requires, from a psychometric perspective, good internal consistency and precision. Internal consistency was studied within the Classical Theory Test framework and precision was assessed by plotting Test Information Curves (TICs), Item Information Curves (IICs) and Standard Error Curves.
5. Functions in the same way in different (sub)groups.This requires, from a psychometric perspective, measurement invariance (or absence of Differential Item functioning [DIF]) between relevant (sub)groups.In this study, we explored DIF for sex (male, female), age (under 53 years, over 53 years; 53 years was the median age of the sample), region (north, east, south, west), educational level (low, middle, high), and ethnicity (native, first and second-generation western immigrant, first and second-generation non-western immigrant). DIF analyses were performed using an ordinal logistic regression framework.
6. Can be used, for international studies, to compare cultural/language groups.This requires, from a psychometric perspective, cross-cultural validity (or absence of DIF) between these groups. In this study, we compared the language groups Dutch and US English, using data from the US PROMIS Wave 1 sample [12, 18]. The PROMIS Wave 1 sample included 21,133 respondents, with 1532 recruited from primary research sites associated with PROMIS network sites and the vast majority (19,601) from YouGovPolimetrix’s panel sample. DIF analysis was performed using a ordinal logistic regression framework (Table 1).

## Results

### Participants

The PROMIS-GH was completed by 4370 Dutch adults from the general population (in 4 samples). Table 2 summarizes the demographic characteristics of the study samples as well as the Dutch general population. The differences in demographic characteristics between our samples and the Dutch general 2016 population, were all less or equal to 2.5% (Table 2).

**Table 2:** Demographic characteristics of the participantsof the total and sub samples of the Dutch general population, and the Dutch general population

Variable	Dutch generalpopulation studytotal sample (N = 4370)		Dutch general population study sample 1 (N = 1052)	Dutch general population study sample 2 (N = 1006)	Dutch general population study sample 3 (N = 1002)	Dutch general population study sample 4 (N = 1310)	Dutch general population 2016^a^ (N = 13,562,539)
	Mean ± SD	Percentage	Percentage	Percentage	Percentage	Percentage	Percentage
*Age (years):*	51.2 ± 16.6						
18–39		32.9	31.7	31.3	31.5	35.1	33.7
40–65		44.1	45.0	45.1	45.6	42.4	43.6
> 65		23.1	23.3	23.6	22.9	22.5	22.7
*Gender:*							
Male		47.3	47.4	47.5	47.6	47.4	49.2
Female		52.7	52.6	52.5	52.4	52.6	50.8
*Educational level:*							
Low		29.1	27.8	27.9	29.3	31.0	30.2
Middle		40.9	40.2	42.3	42.6	39.8	40.2
High		30.0	32.0	29.8	28.0	29.2	29.6
*Region:*							
North		10.2	9.9	10.9	10.2	9.4	10.2
East		20.5	21.4	19.8	19.9	20.8	20.8
South		21.2	21.1	21.9	20.1	20.5	21.6
West		47.9	47.1	47.1	49.6	49.2	47.4
Missing		0.3	0.5	0.3	0.3	0.2	-
*Ethnicity:*							
Native		78.2	76.7	79.1	77.2	79.5	78.6
First and second generation western immigrant		11.8	11.7	11.9	12.7	11.8	10.3
First and second generation non-western immigrant		10.0	11.6	9.0	10.1	8.7	11.2
Global Mental Health (GMH) T-score^b^	44.7 ± 8.0						
Global Physical Health (GPH) T-Score^b^	45.2 ± 9.2						

N, number; SD, standard deviation

^a^Based on data from statistics Netherlands (http://www.cbs.nl)

^b^T-scores were calculated using Scoring Service from Assessment Center

### Items

Table 3 reports the results of the item descriptive statistics. The highest (better) scoring category was chosen by 51.4%, 24.6%, and 23.6% forGlobal06 (physical function), Global07 (pain intensity), and Global10 (emotional problems), respectively (Table 3).

**Table 3:** PROMIS Global Health items descriptives, scalability, graded response model fit, range of item discrimination and difficulties, internal consistency, measurement invariance and cross-cultural validity in the total sample of the Dutch general population (N = 4370)

Items description	Descriptives				Mokken scalability	GRM model fit		Range of item discrimination and difficulties*					Internal consistency		Measurement invariance					Cross cultural validity
	Mean (SD)	Range (Min–Max)	Skewness (SE)	Kurtosis (SE)	H_i_ (SE)	S-X^2^	p	α	β1	β2	β3	β4	IIT	αIID	Sex	Age	Region	Educ. level	Ethnicity	
*Global Mental Health*																				
Global02: quality of life	3.0 (0.9)	1–5	0.08 (0.04)	− 0.17 (0.07)	0.633 (0.009)	94.550	** < 0.001**	3.507	− 1.935	− 0.603	0.710	1.893	0.72	0.76	0.0025	0.0065	0.0005	0.0014	0.0001	0.0041
Global04: mental health, including mood and ability to think	3.1 (1.0)	1–5	0.01 (0.04)	− 0.32 (0.07)	0.654 (0.009)	160.820	** < 0.001**	2.757	− 2.047	− 0.783	0.529	1.648	0.73	0.75	0.0007	0.0031	0.0001	0.0006	0.0011	0.0028
Global05: satisfaction with social activities and relationships	3.0 (0.9)	1–5	0.03 (0.04)	− 0.15 (0.07)	0.603 (0.010)	87.731	** < 0.001**	2.849	− 1.855	− 0.644	0.739	1.916	0.68	0.78	0.0017	0.0031	0.0003	0.0012	0.0018	0.0032
Global10: bothered by emotional problems	3.7 (1.0)	1–5	− 0.31 (0.04)	− 0.64 (0.07)	0.518 (0.012)	323.387	** < 0.001**	1.321	− 3.717	− 1.779	− 0.220	1.181	0.52	**0.85**	0.0070	0.0165	0.0017	0.0034	0.0020	0.0136
*Global physical health*																				
Global03: physical health	2.7 (0.9)	1–5	0.24 (0.04)	− 0.23 (0.07)	0.565 (0.010)	145.562	** < 0.001**	2.266	− 1.792	− 0.239	1.094	2.248	0.62	0.71	0.0010	0.0022	0.0019	0.0021	0.0041	0.0046
Global06: carry out every day physical activities	4.1 (1.2)	1–5	− 0.91 (0.04)	− 0.32 (0.07)	0.525 (0.013)	184.359	** < 0.001**	1.943	− 2.668	− 1.619	− 0.705	− 0.055	0.55	0.74	0.0018	0.0013	0.0005	0.0030	0.0003	0.0006
Global07: pain on average	3.1 (2.7)	1–5	0.41 (0.04)	− 1.16 (0.07)	0.547 (0.010)	82.367	** < 0.001**	2.063	− 3.641	− 1.306	− 0.279	0.916	0.62	0.71	0.0013	0.0047	0.0002	0.0013	0.0011	0.0048
Global08: fatigue on average	3.4 (1.0)	1–5	− 0.16 (0.04)	− 0.50 (0.07)	0.513 (0.011)	64.189	** < 0.001**	1.705	− 2.844	− 1.291	0.144	1.548	0.56	0.74	0.0029	**0.0458**	0.0018	0.0049	0.0034	0.0002
*Items not contributing to the subscale scores*																				
Global01: general health	2.8 (0.9)	1–5	0.20 (0.04)	− 0.16 (0.07)	–	–	–	–	–	–	–	–	–	–	–	–	–	–	–	–
Global09: usual social activities and roles	3.0 (0.9)	1–5	0.07 (0.04)	− 0.12 (0.07)	–	–	–	–	–	–	–	–	–	–	–	–	–	–	–	–
Recommended values	N/A	N/A	N/A	N/A	> 0.30	N/A	≥ 0.001	> 1.0	N/A	N/A	N/A	N/A	≥ 0.40	< α	< 0.02	< 0.02	< 0.02	< 0.02	< 0.02	< 0.02

α, Cronbach’s alpha; αIID, α if item deleted GRM, Graded Response Model; Hi, scalability coefficient for item; IIC, itam-to-total correlation; N/A, not applicable; p, *p*-value; S-X^2^, item fit statistics under the Graded Response Model; SD, standard deviation; SE, standard error

Statistics values beyond the recommended cut-offs presented in bold

For interpretation of the indexes, refer to Table 1

Global08 and Global10 have been recoded according the instruction in the PROMIS-GH Scoring Manual

Possible response range for each item varies from 1 to 5 points

Cross-cultural validity was studied using data from the US PROMIS Wave 1 sample, obtained from the Health Measures Dataverse [12, 18]

* Item parameters were estimated using the Dutch dataset in this paper; the official PROMIS item parameters used in CAT are available from help@healthmeasures.net

### Is it legitimate to calculate IRT-based scores for PROMIS-GH?

*Dimensionality.* The CFA on the entire PROMIS-GH highlighted some departure from the two-factor structure (Comparative Fit Index [CFI] = 0.95, Tucker Lewis Index [TLI] = 0.92,Root Mean Square Error of Approximation [RMSEA] = 0.16,Standardized Root Mean Square Residual [SRMR] = 0.07). The Exploratory Bifactor Analysis did not converge for GMH without restricting the number of group factors since loadings > 1 were present. After fixing the number of group factors to 2 and, consequently, constraining the general factor loadings to avoid specifying an under-identified model, the model converged. The Explained Common Variance (ECV) revealed the presence of a strong general factor (ECV = 0.80) whereas the Omega-Hierarchical (ωH) did not met the criterion (ωH = 0.75). Similarly, the Exploratory Bifactor Analysis performed on the GPH revealed that the general factor explained most of the common variance (ECV = 0.71) whereas the ωH did not met the criterion (ωH = 0.65) (Table 1). The unidimensional CFAs, run on each subscale separately, revealed that the all the fit indices, except for the RMSEA, supported adequate fit (GMH: CFI = 0.98, TLI = 0.95, RMSEA = 0.22, SRMR = 0.04; GPH: CFI = 0.99, TLI = 0.97, RMSEA = 0.12, SRMR = 0.03) (Table 1). Finally, Pearson’s correlation coefficients between the raw and IRT-based score were 0.985 and 0.988 (*p* < 0.001) for GMH and GPH, respectively, and Pearson’s correlation coefficients between the GMH and GPH were 0.561 and 0.562 (*p* < 0.001) for raw and IRT-based scores, respectively.

*Local dependence.* No local dependence was detected (all residual correlations between items < 0.20) (Table 1).

*Monotonicity.* The scalability coefficients for the scales were high (H = 0.60 for GMH, and 0.54 for GPH) (Table 1). The scalability coefficients of the items were above the recommended cut-off (H_i_ > 0.30) (Table 3). Moreover, visual inspection of the Mokken scale Item Characteristic Curves (ICCs) showed that none of the items presented violations to monotonicity (Fig. 1). Global06 presented the lowest distance between the thresholds; Additional file 1: Figure S1 presents a detail of the Global06 ICC that confirms that none of its thresholds are disordered.

*IRT-model fit.* Both subscales fitted the GRM model (RMSEA = 0.03 for GMH, and 0.02 for GPH). However, all items displayed misfit to the GRM model (*p* < 0.0001) (Table 3). To avoid flagging items with negligible (i.e., as a consequence of excessive power) misfit, 10 mutually exclusive random samples of 473 subjects each were created and the item fit to the GRM model was computed in each sample; moreover, in order to adjust for type-I errors we used a Bonferroni-corrected *p*-value of 0.000625 (i.e., 0.05/80 comparisons). The ten IRT-analyses showed satisfactory item fit statistics for all items (*p* ≥ 0.001) except for Global02 (overall quality of life), Global04 (mental health), and Global05 (satisfaction with social activities) (*p* < 0.001 in Sample#5, Sample#8, and Sample#3) and Global10 (emotional problems) (*p* < 0.001 in Sample#1, Sample#3, Sample#6, Sample#7, Sample#8, and Sample#9) for GMH, and Global07 (pain intensity) (*p* < 0.001 in Sample#5) for GPH (Table 4). Empirical plots of the items displaying unsatisfactory fit statistics in at least one subsample were inspected (Additional file 2: Figure S2-S3). Only Global10 showed non-negligible misfit.

**Table 4:** Fit statistics of the PROMIS Global Mental Health and Global Physical Health items in ten random sub samples of the Dutch general population (N = 437 per sample)

Items	Sample 1		Sample 2		Sample 3		Sample 4		Sample 5		Sample 6		Sample 7		Sample 8		Sample 9		Sample 10	
	S-X^2^	p.S-X^2^	S-X^2^	p.S-X^2^	S-X^2^	p.S-X^2^	S-X^2^	p.S-X^2^	S-X^2^	p.S-X^2^	S-X^2^	p.S-X^2^	S-X^2^	p.S-X^2^	S-X^2^	p.S-X^2^	S-X^2^	p.S-X^2^	S-X^2^	p.S-X^2^
*Global mental health*																				
Global02	12.559	0.084	13.954	0.083	6.435	0.696	6.796	0.658	42.118	**0.000**	10.239	0.509	22.968	0.003	8.201	0.414	14.972	0.133	8.879	0.449
Global04	14.218	0.115	21.793	0.040	17.267	0.100	18.825	0.043	34.371	0.001	30.387	0.007	24.238	0.007	39.123	**0.000**	23.835	0.021	18.996	0.089
Global05	18.809	0.027	12.457	0.189	30.303	**0.000**	11.846	0.540	8.327	0.684	17.109	0.105	4.854	0.847	25.040	0.015	19.845	0.031	14.416	0.155
Global10	63.991	**0.000**	39.085	0.007	43.508	**0.000**	35.477	0.003	42.483	0.001	56.640	**0.000**	47.465	**0.000**	73.273	**0.000**	47.167	**0.000**	19.519	0.191
*Global physical health*																				
Global03	23.068	0.041	30.304	0.003	17.584	0.092	28.022	0.009	25.689	0.019	24.152	0.030	9.089	0.614	19.221	0.057	14.474	0.341	25.292	0.046
Global06	23.940	0.121	25.469	0.085	16.071	0.519	22.376	0.171	27.245	0.099	15.739	0.471	17.621	0.347	28.619	0.038	26.008	0.074	22.647	0.123
Global07	19.943	0.174	15.422	0.350	13.859	0.460	16.168	0.303	38.288	**0.000**	11.051	0.854	24.764	0.053	39.554	0.001	12.297	0.503	14.859	0.388
Global08	7.800	0.856	17.086	0.449	18.065	0.204	20.637	0.193	13.724	0.687	20.874	0.141	16.816	0.536	32.616	0.019	19.606	0.143	27.718	0.048
Recommended values	N/S	≥ p-BC	N/S	≥ p-BC	N/S	≥ p-BC	N/S	≥ p-BC	N/S	≥ p-BC	N/S	≥ p-BC	N/S	≥ p-BC	N/S	≥ p-BC	N/S	≥ p-BC	N/S	≥ p-BC

S-X^2^, item fit statistics under the Graded Response Model; p.S-X^2^, p-value related to the S-X^2^; p-BC, *p*-value Bonferroni corrected

*p*-value was set at 0.000625 (Bonferroni corrected)

Statistics values beyond the recommended cut-off presented in bold

### Is PROMIS-GH able to discriminate between different levels of the construct/trait?

*Range of item discrimination.* Item slope parameters varied from 1.3 to 3.5 for GMH, and from 1.7 to 2.2 for GPH (Table 3).

### Does the PROMIS-GH cover the relevant range of the construct/trait?

*Range of item difficulties.* Item threshold parameters ranged between − 3.7 and 1.9 for GMH, and between − 3.6 and 2.2 for GPH (Table 3).

### Is the PROMIS-GH measure reliable?

*Internal consistency.* The Cronbach’s alpha was sufficient for GMH (0.83), and GPH (0.78). Alpha values after item deletion decreased for all items, except forGlobal10 (emotional problems). Finally, corrected item-to-total correlations were satisfactory for all items of both subscales (r_s_ > 0.40) (Table 1).

**Fig. 2:**
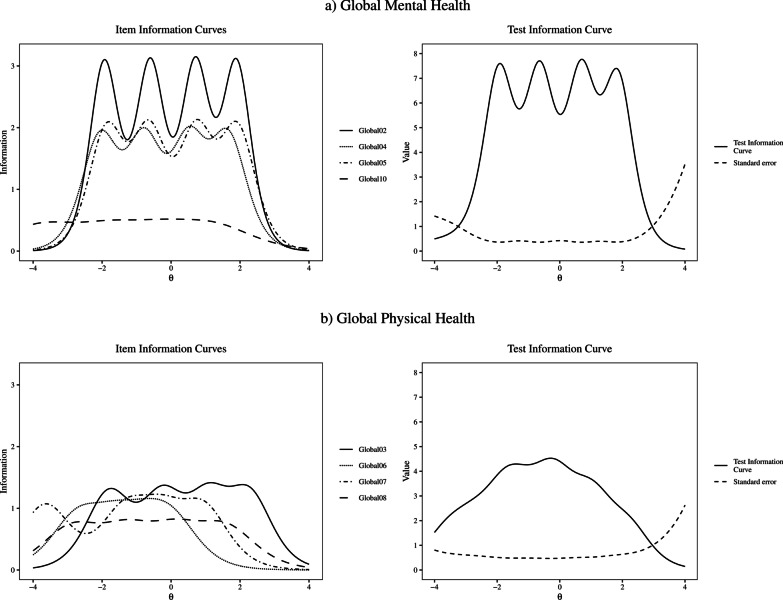
Item and Test Information Curves of the 4-item Global Mental Health and the 4-item Global Physical Health (N = 4370)

*Precision.* Figure 2 displays the IICs and the TICs. The total score information was high across the latent trait for both subscales. However, the IICs forGlobal10 (emotional problems) was low; indeed, this item presented low information in most portions of the latent trait but provided more information than the other items at very low latent trait values (Fig. 2).

### Do PROMIS-GH items function in the same way in different (sub)groups?

**Fig. 3:**
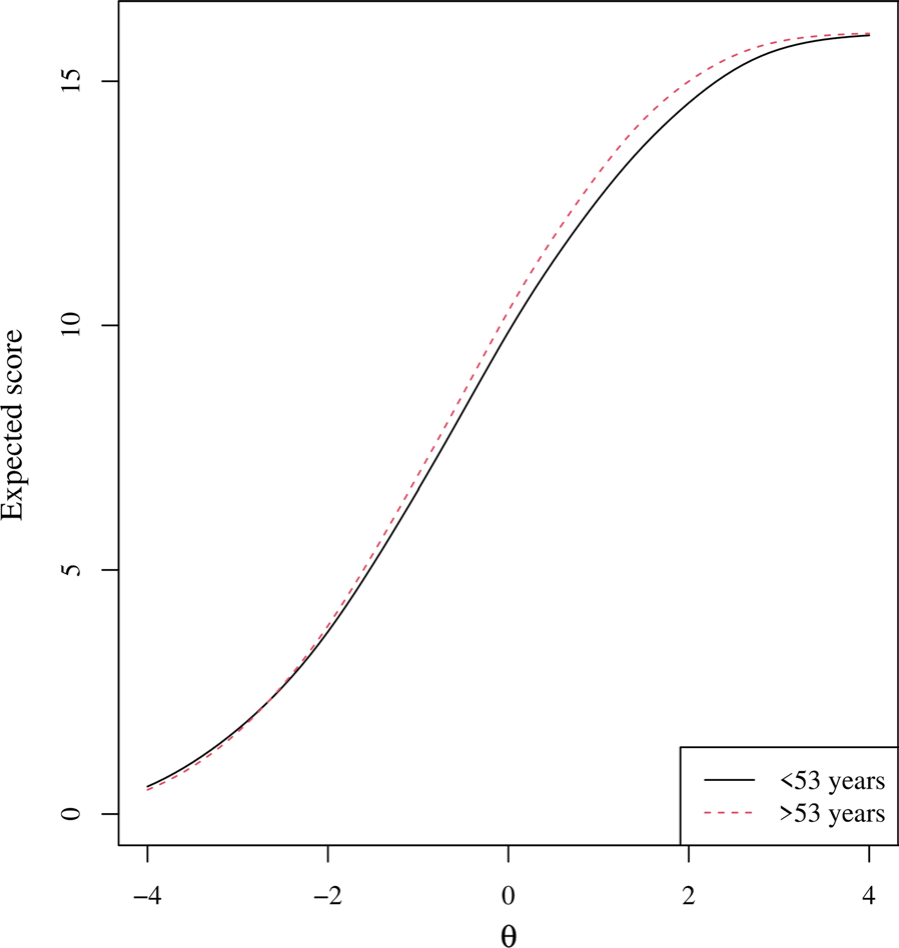
The overall impact of Differential Item Functioning of Global08 (fatigue) for age on the Test Characteristic Curve (TCC). The TCC shows the relation between the total item scores (y-axis) and theta (x-axis) (N = 4370)

*Measurement invariance.* None of items presented DIF for gender, region, educational level and ethnicity (Table 3). Only Global08 (fatigue) showed non-negligible DIF for age (McFadden’s pseudo R^2^ change between model 1 and 2 = 0.0458 and between model 2 and 3 = 0.0015), with younger participants being more likely to endorse lower response categories than older participants at the same level of fatigue. However, after visual inspection of the Test Characteristic Curves per group, it was concluded that the impact of DIF on the total score was negligible (Fig. 3).

*Cross-cultural validity.* Cross-cultural validity was supported, as no DIF for language was detected (Table 3).

## Discussion

This is the first study evaluating the psychometric properties of the PROMIS-GH outside of the US. We found sufficient evidence for structural validity of the GPH subscale. However, structural validity of the GMH subscale could be improved as Global10 (emotional problems) showed misfit to the IRT-model in six out of 10 (60%) subsamples. Moreover Global10 (emotional problems) had the lowest item-scale correlation, was the only item that would increase Cronbach’s alpha if deleted, had the lowest discrimination parameter and lowest information value. Sufficient internal consistency, measurement invariance (except Global08 [fatigue] for age) and cross-cultural validity were found.

The analysis of the dimensionality of the PROMIS-GH showed that considering the GMH and the GPH as unidimensional scales might be the most appropriate strategy. The use of a multidimensional model was ruled out by our 2-factor model, the results of which are comparable to the 2-factor model results of Hays et al. [13] and Katzan and Lapin [14] (RMSEA = 0.11). The exploratory factor analysis showed that most of the variance in the responses to both subscales is explained by general factors, and this supports the use of unidimensional models. The fact that the RMSEA values of the unidimensional CFA models were above the cut-off does not invalidate this choice. In previous studies, many other PROMIS measures have also shown high RMSEA values under CFA [27–31]. According to Cook et al. [32], traditional cut-off for CFA fit statistics are not suitable for assessing unidimensionality of item banks measuring latent health variables. Reise et al. [33] reported that the RMSEA statistic may be problematic for assessing unidimensionality of latent health traits, and they suggested that the SRMR, as well as the ECV and omega H computed through a bifactor analysis, might be more appropriate to determine whether an instrument is “unidimensional enough” and, as a consequence, if IRT parameters computed assuming an unidimensional model are not biased. The SRMR values (SRMR = 0.04 for GMH and 0.03 for GPH) indicated a good fit to the model. The Explanatory Bifactor Analysis revealed that the ECV values met the criterion, but omega H values were below the recommended threshold. Taken together, these analyses support the use of separate unidimensional models for the GMH and the GPH.

Although the global fit to the GRM model was adequate, some items displayed lack of fit after adjusting for Type I errors. The misfit of items Global07, Global02, Global04 and Global05, however, was present in no more than 3 random subsamples, and visual inspection of their empirical plots revealed only slight deviations from the expected item response functions. On the contrary, item-level misfit of Global10 was apparent in most of the random subsamples and by visual inspection of its empirical plot. Lack of fit to the GRM model might result in biased ability and item parameters estimates [34]. Therefore, the parameters of item Global10 should be interpreted with caution.

It is possible that these subscales do not perfectly fit the IRT-model, because they do not measure a real psychometric construct (they do not form a reflective, but rather a formative model). This has an impact on the requirement of unidimensionality and calculation and interpretation of scores. A formative model means that measured variables are considered to be the cause of the construct (for example like the Apgar score, which is defined by its components); on the other hand, in the reflective model, the indicators are considered to be caused by that construct (for example, an instrument measuring anxiety) [35, 36]. In the case of the PROMIS-GH, it could be argued that its items can be seen as aspects that define global health, rather than being manifestations of it (e.g., overall quality of life, mental health, satisfaction with social activities and emotional problems define global mental health and are not its manifestations); that changes in the items would change global health rather than vice versa; and that dropping one item would alter the domain the construct [37]. If these scales are considered as a formative model, unidimensionality of the scales is not required. The total score can be calculated by the sum of the responses to each item. A higher score means that more aspects of global health are affected. On the other hand, the items in these scales could be considered as manifestations of global health (reflective model). In that case, the scales should be unidimensional and IRT-based scoring can be used. A higher score means better global health. This is the current assumption of how the PROMIS-GH is being used. Since the correlations between the raw scores and the IRT-based scores are high (r = 0.985 and 0.988 for GMH and GPH, respectively), it seems appropriate to use IRT-based scoring even if the scales do not perfectly fit the IRT-model. A further advantage of IRT-based scoring is that interval scores allows the correct use of parametric statistics [38, 39]. Moreover, interval measurements showed a greater magnitude of changes when compared to raw scores [40, 41]; consequently the results of clinical trials using raw scores could lead to incorrect conclusions [39, 42]. Finally, the PROMIS initiative uses interval scores by default and these scores can easily be estimated on their website.

The results of the monotonicity analysis showed that no items presented disordered thresholds. Upon a visual inspection of the ICCs, only the Global06 showed a short interval in the thresholds between 3 (Moderately) and 4 (Mostly) scores, and between 4 (Mostly) and 5 (Completely) scores. This result may be due to the content of the response options; indeed, Global06 is the only item that has these response categories. Our subjects may had difficulty discriminating the fine differences between these three categories. However, the findings of the Mokken scale analysis confirmed that Global06 presented monotonicity (H_i_ = 0.525). Therefore, in light of these results, we do not suggest a modification of the Global06 response categories.

Our results show that the item slope parameters (discriminative ability) of each item is higher than the cut-off of 1.0; this means that each item is able to distinguish different levels of latent traits that it intends to measure. On the other hand, there is no range of interpretations for the difficulties of the items; the range should be as wide as possible; our results showed a wide range for both GMH and GPH which suggests that each subscale is able to measure a large range of the latent variable it intends to measure.

Most of PROMIS-GH items function in the same way across different groups, as indicated by measurement invariance, which means that the same IRT-model can be applied to compare different groups of patients in terms of gender, educational level and ethnicity and to compare US versus Dutch patients. Our results are similar to those of the previous literature. A recent study [43] found no DIF in any GMH and GPH items across age groups, medical or clinical complexity environment in 7964 subjects. For Dutch and Flemish users, the Dutch-Flemish Assessment Center offers real-time IRT-based scoring of the PROMIS-GH (using the same algorithm as Scoring Service) for use in clinical practice, through a software link with several data collection platforms.

Our results showed that Global10 (emotional problems) showed problems with item fit and precision,. Similar results were reported by Hays et al. [13] who found thatGlobal07 (pain intensity), Global08 (fatigue) andGlobal10 (emotional problems) had the lowest item information. However, Global10 (emotional problems) showed a good corrected item-to-total correlation, is more informative than the other items at the very low end of the scale (i.e., worst mental health), measurement invariance and its cross-cultural validity were supported. The Global10 content could be the cause of its problems highlighted by our analyses; indeed, Global10 investigates both the presence of emotional problems (i.e., anxiety and depression) and their bothersomeness (i.e., how much the patient perceives their presence negatively). A low score could indicate that the patient has no emotional problems (and therefore cannot be bothered), or that the patient perceives emotional problems, but is not bothered about it. The Cronbach’s alpha increased after item deletion, which could indicate that the responses to this item have some irrelevant variance for the construct. However, emotional problems are important health problems for many patients; therefore removing this item would reduce content validity. Therefore, we think it is justifiable, at this stage, to maintain the item in the scale. Maybe the problems arises from the reversed scoring. However, if future studies consistently will show Global10 (emotional problems)to be the poorest performing item, replacing this item with another emotional health item in the GMH subscale could be considered. Hence, for now, we recommend to use the GMH scale as it is.

The strength of this study concerns the large number of enrolled participants answering the PROMIS-GH. However, this study also has limitations that deserve to be discussed. Unfortunately, response rate information is not available. Moreover, we studied subjects from the general population that may include not many patients seen in daily clinical practice, although it seems fair to assume that the general population also includes people with different diseases. Also, our analyses were conducted using a convenience sample of Dutch–speaking adults; this issue could limit the generalizability of the results to other contexts. Since this is one of the most commonly used PROMIS measures, recommended by ICHOM to be used in clinical practice, future studies in clinical populations and other countries are recommended. Finally, in order to study the item ability to discriminate between different levels of the construct, and, consequently, its ability to measure change within person over time, we assessed the item discrimination; test–retest reliability and responsiveness are more relevant to measure change over time; therefore, future researches should assess these psychometric properties.

Our results and those of other articles [13, 14] displayed limitations of the factor structure of GMH, which was to be expected considering the breath of the mental health construct. Global10 (emotional problems) showed misfit to the IRT-model, but its content validity and its information value suggests to maintain this item. Future content validity studies, involving patients, might further explore this issue in order to confirm our suggestion to keep the Global10 (emotional problems). Nevertheless, our findings provide support for the structural validity (including IRT-model fit), internal consistency, measurement invariance, and cross-cultural validity of PROMIS-GH in the Dutch general population. Given the lack of studies on the PROMIS-GH, we consider our results preliminary. Only if future studies confirm our results, a decision on structural GMH modifications should be taken into account. Hence, our results can be considered good enough for using the GMH and GPH scales in their current form.

## Conclusion

Our findings showed that the psychometric properties of the PROMIS-GH in a large Dutch sample are acceptable. Sufficient local independence, monotonicity, GRM fit, internal consistency, measurement invariance and cross-cultural validity were found. However, that Global10 (emotional problems), showed problems with item fit and precision. If future studies confirm our results, the measurement properties of GMH could be improved by modifying or replacing Global10.

## Supplementary Information

**Additional file 1: Figure S1.** Detail of the Item Characteristics Curve for Global 06.

**Additional file 1: Figure S2-S3.** Empirical plot of items display misfit to the IRT model in at least one subsample.

## Data Availability

The data is available from the corresponding author on reasonable request.
