# Supplementary material for: Psychometric properties of the patient-reported outcomes measurement information system scale v1.2: global health (PROMIS-GH) in a Dutch general population
Source: Health Qual Life Outcomes. 2021 Sep 27;19:226. doi: 10.1186/s12955-021-01855-0 (PMC8477486; doi:10.1186/s12955-021-01855-0)
Supplement: Supplementary file 1 — Additional file 1: Figure S1. Detail of the Item Characteristics Curve for Global 06. [file 12955_2021_1855_MOESM1_ESM.docx]

**Figure S1.** Detail of the Item Characteristic Curves for Global06

Note. The line representing the probability to endorse the 4^th^ response category, albeit for a very short interval, is above the lines representing the probability to endorse the 3^rd^ and 5^th^ response categories. Therefore, the thresholds of Global06 are ordered.

**
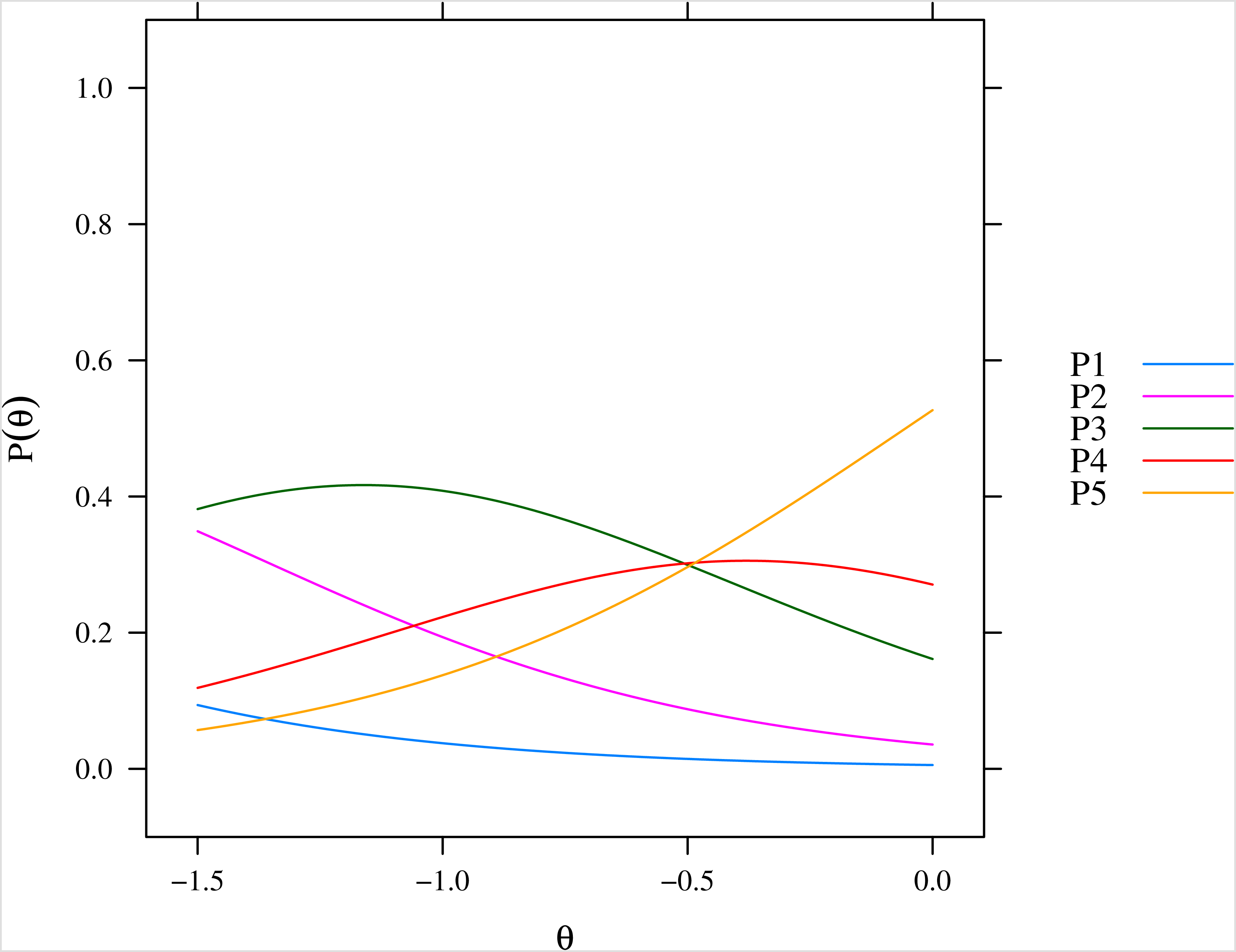
**
