# Supplementary material for: Psychometric properties of the patient-reported outcomes measurement information system scale v1.2: global health (PROMIS-GH) in a Dutch general population
Source: Health Qual Life Outcomes. 2021 Sep 27;19:226. doi: 10.1186/s12955-021-01855-0 (PMC8477486; doi:10.1186/s12955-021-01855-0)

**Figure S2**. Empirical plot of the Global07 item of the Global Physical Health subscale.


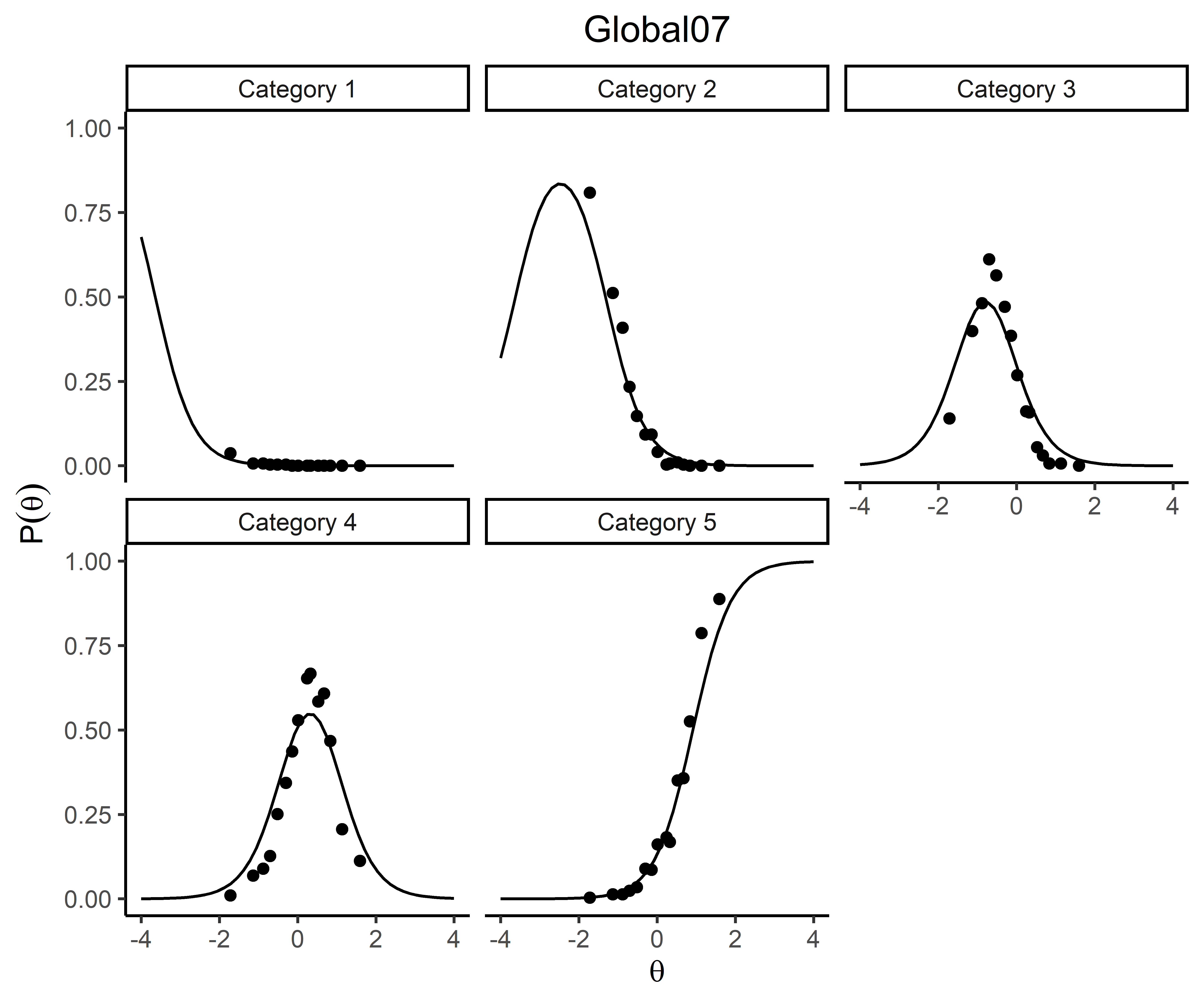
Note. The solid line represents the expected item response function based on the GRM model. Dots represent observed probability of endorsing each category by groups of participants with similar latent trait across the θ continuum. The plot is drawn using data from the total sample (n=4,370)

**Figure S3.** Empirical plots of the Global02, Global04, Global05 and Global06 items of the Global Mental Health subscale.

Note. The solid line represents the expected item response function based on the GRM model. Dots represent observed probability of endorsing each category by groups of participants with similar latent trait across the θ continuum. The plots are drawn using data from the total sample (n=4,370).


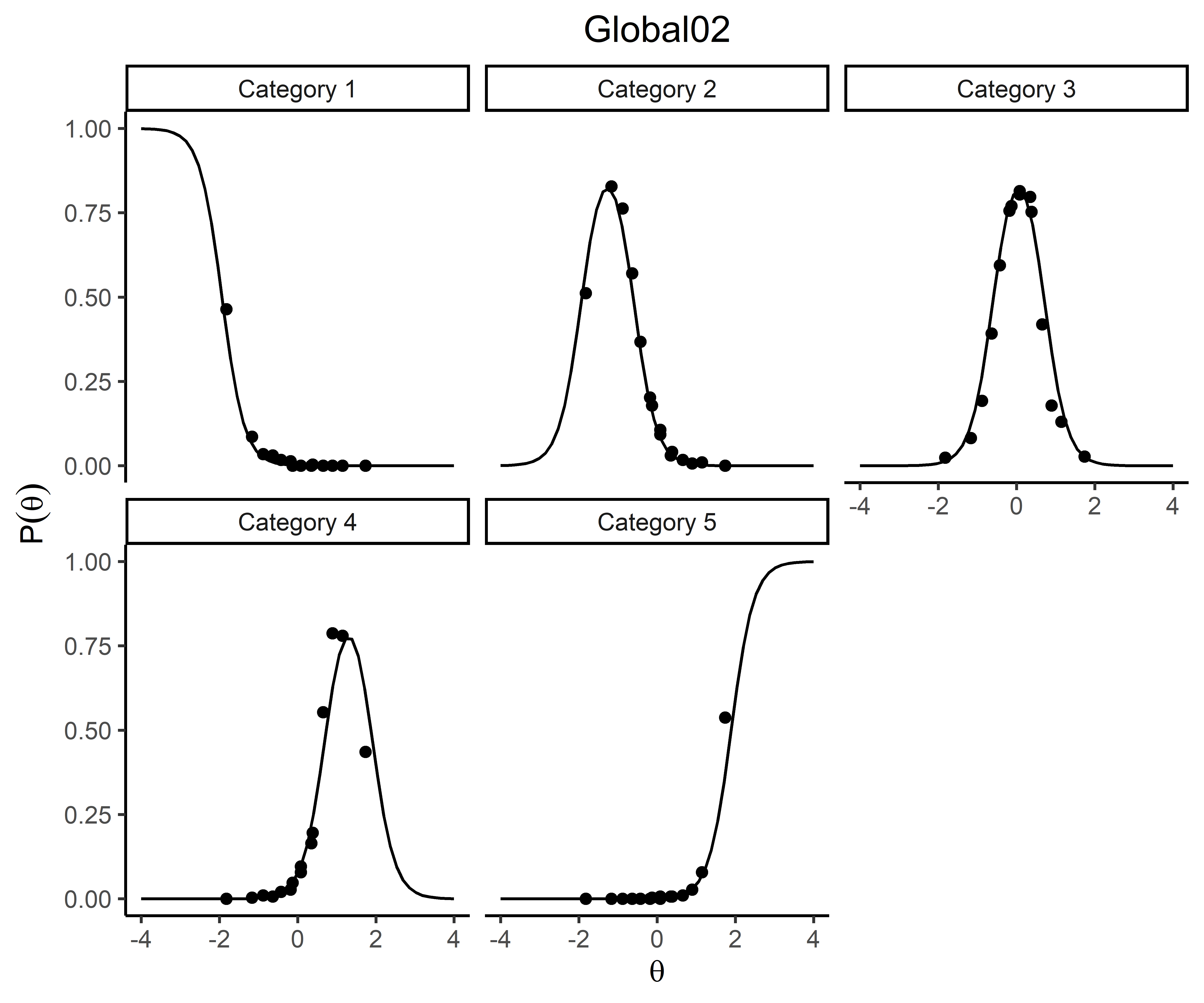

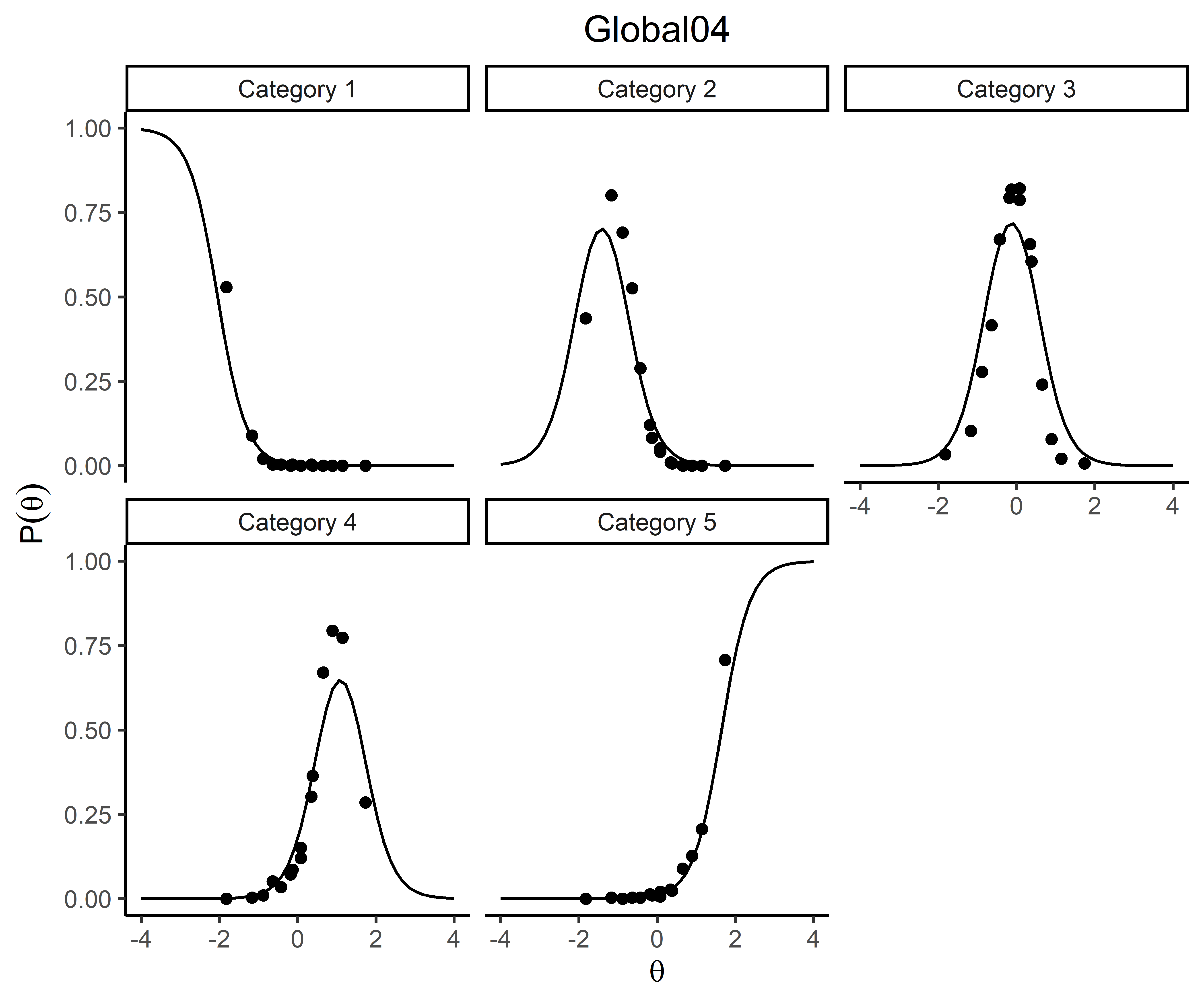

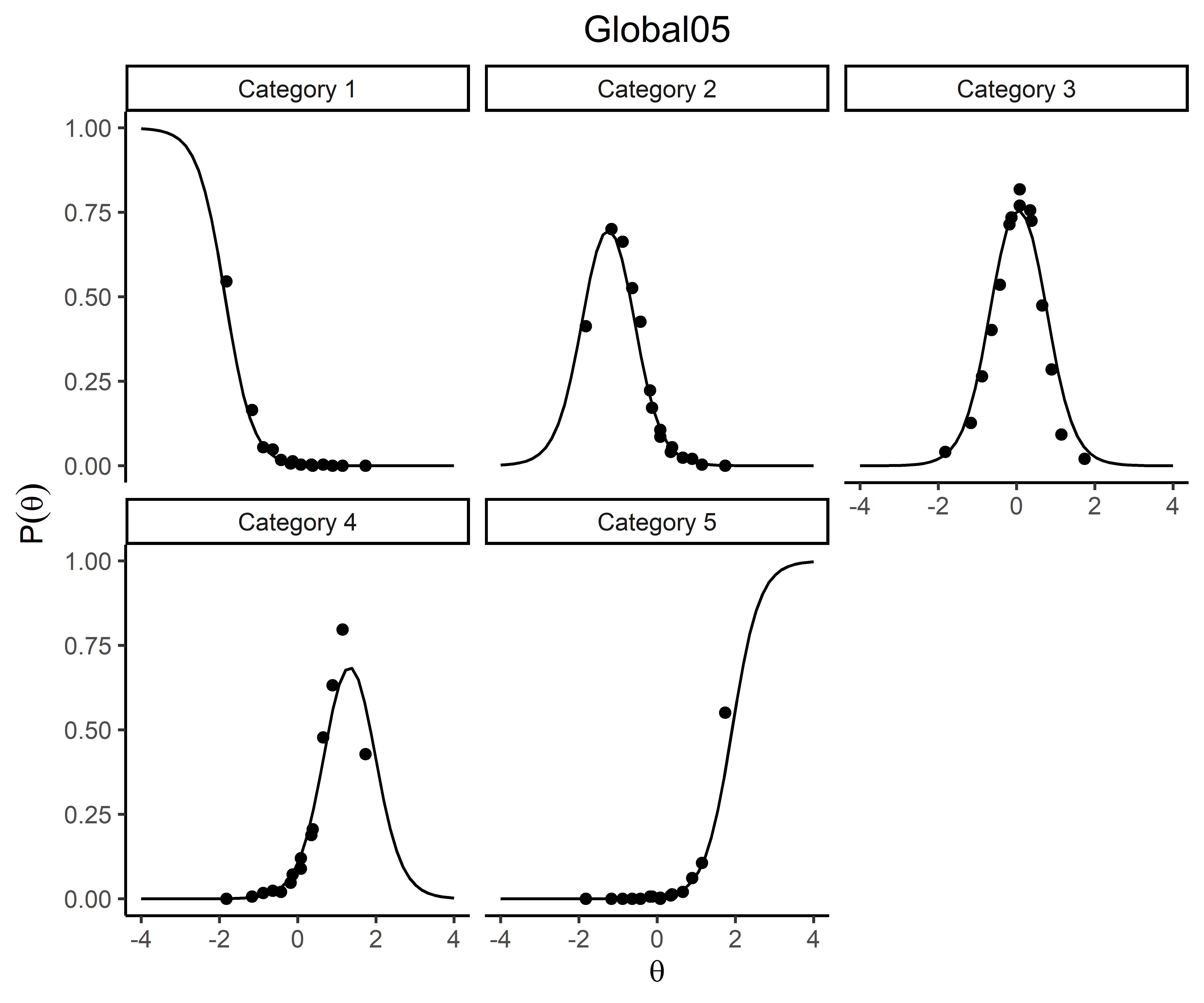

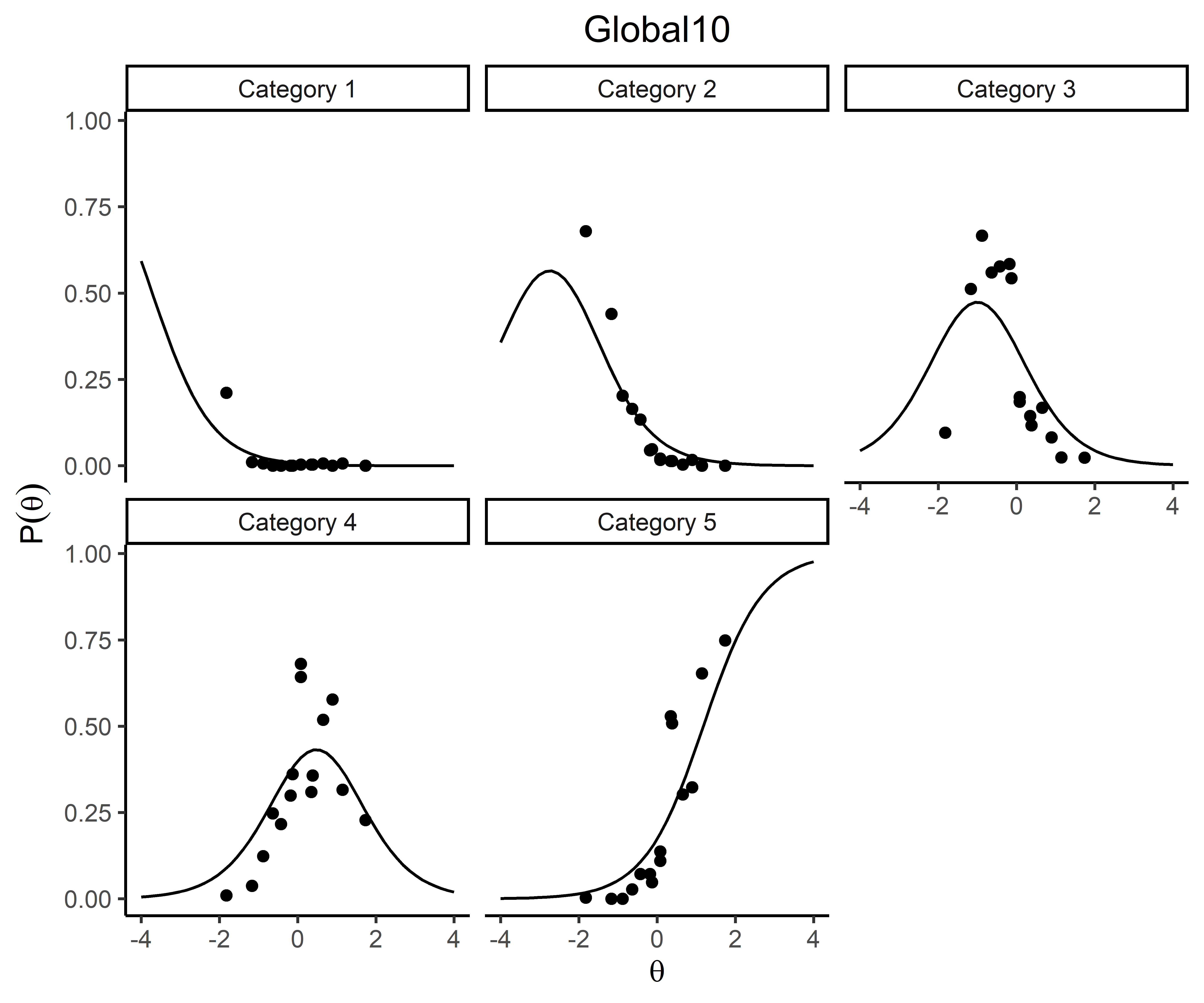

Supplement: Supplementary file 2 — Additional file 1: Figure S2-S3. Empirical plot of items display misfit to the IRT model in at least one subsample. [file 12955_2021_1855_MOESM2_ESM.docx]
